# (Cost)-effectiveness of a multi-component intervention for adults with epilepsy: study protocol of a Dutch randomized controlled trial (ZMILE study)

**DOI:** 10.1186/s12883-014-0255-3

**Published:** 2014-12-24

**Authors:** Loes AM Leenen, Ben FM Wijnen, Reina JA de Kinderen, Marian HJM Majoie, Caroline M van Heugten, Silvia MAA Evers

**Affiliations:** CAPHRI School for Public Health and Primary Care, Maastricht University, P.O. Box 616, 6200 MD Maastricht, the Netherlands; Department of Health Services Research, Maastricht University, Duboisdomein 30, 6229 GT Maastricht, the Netherlands; Department of Research & Development, Epilepsy Centre Kempenhaeghe, Heeze, the Netherlands; Department of Neurology, Academic Centre for Epileptology, Epilepsy Centre Kempenhaeghe & Maastricht University Medical Centre, Maastricht, The Netherlands; MHENS, School for Mental Health and Neuroscience, department of Psychiatry and Neuropsychology, Maastricht University Medical Centre, Maastricht, the Netherlands; Department of Neuropsychology and Psychopharmacology, Maastricht University, Maastricht, the Netherlands; Trimbos Institute, Netherlands Institute of Mental Health and Addiction, Utrecht, the Netherlands

## Abstract

**Background:**

In patients with epilepsy, poor adherence to anti-epileptic drugs has been shown to be the most important cause of poorly controlled epilepsy. Furthermore, it has been noted that the quality of life among patients with epilepsy can be improved by counseling and treatments aimed at increasing their self-efficacy and concordance, thus stimulating self-management skills. However, there is a need for evidence on the effectiveness of such programs, especially within epilepsy care. Therefore, we have developed a multi-component intervention (MCI) which combines a self-management/education program with e-Health interventions. Accordingly, the overall objective of this study is to assess the (cost)-effectiveness and feasibility of the MCI, aiming to improve self-efficacy and concordance in patients with epilepsy.

**Methods:**

A RCT in two parallel groups will be conducted to compare the MCI with a control condition in epilepsy patients. One hundred eligible epilepsy patients will be recruited and allocated to either the intervention or control group. The intervention group will receive the MCI consisting of a self-management/education program of six meetings, including e-Health interventions, and will be followed for 12 months. The control group will receive care as usual and will be followed for 6 months, after which patients will be offered the possibility of participating in the MCI. The study will consist of three parts: 1) a clinical effectiveness study, 2) a cost-effectiveness study, and 3) process evaluation. The primary outcome will be self-efficacy. Secondary outcomes include adherence, side effects, change in seizure severity & frequency, improved quality of life, proactive coping, and societal costs. Outcome assessments will be done using questionnaires at baseline and after 3, 6, 9, and 12 months (last two applicable only for intervention group).

**Discussion:**

In times of budget constraints, MCI could be a valuable addition to the current healthcare provision for epilepsy, as it is expected that higher concordance and self-efficacy will result in reduced use of healthcare resources and an increased QOL. Accordingly, this study is aimed helping patients to be their own provider of health care, shifting epilepsy management from professionals to self-care by patients equipped with appropriate skills and tools.

**Trial registration number:**

NTR4484.

## Background

Epilepsy is a chronic disorder of the brain, characterized by recurrent seizures. Seizures are the result of sudden, excessive electrical discharges in a group of brain cells. Different parts of the brain can be the site of such discharges, resulting in a variety of clinical manifestations [1].

Epilepsy has a considerable psychological and emotional impact, which is strongly reflected in a reduced quality of life (QOL) for patients suffering from epilepsy [2,3]. Living with seizures is likely to affect patients’ daily activities, as it interferes with many aspects of everyday life. Furthermore, epilepsy has been shown to have a large economic impact on society as a whole [4,5]. For example, the unemployment rate among epileptic patients is at least twice as high as in the general population [6-8].

Recent studies have shown that up to 70-80% of newly diagnosed epilepsy patients can be treated successfully (i.e. seizures completely controlled) with anti-epileptic drugs (AEDs) [9-11] and it is estimated that currently more than 80,000 patients in the Netherlands are treated with AEDs [12]. However, to achieve and maintain successful seizure control, adherence to treatment is of major importance. A systematic review argued that effective ways of helping people follow medical treatments could have far larger effects on health than any treatment itself [13]. In addition, it has been recommended that the cost-effectiveness of adherence interventions should be a research priority in the field of chronic diseases [14]. In epilepsy, poor adherence has been shown to be the most important cause of poorly controlled epilepsy [15]. However, this study is striving to improve ‘concordance’ and not only ‘adherence’. The crucial difference is that ‘adherence’ describes only the extent to which a patient takes antiepileptic drugs as prescribed with respect to dosage and dosing intervals [16], while ‘concordance’ includes a consensual agreement about taking AEDs that has been established between patient and practitioner [17].

Concordance with medical treatment is closely linked with the patients’ ability to self-manage their disease, and the latter is shown to be an important factor in determining quality of life [18]. Self-management programs focus on supporting patients in coping with their chronic condition, eventually to maximize quality of life [19], and have been identified as useful for individuals with chronic conditions such as asthma, heart disease, diabetes [19,20]. However, due to several reasons, results of studies relating to chronic patient groups cannot be generalized to patients with epilepsy. For example, the consequences of poor self-management, i.e. not taking AEDs or irregular sleeping patterns, are not always directly observable; seizure deregulation can appear the same day or a couple of days later. Hence, the direct link between poor disease management and the frequency of seizures is not transparent for epilepsy patients. A recent study showed that many patients with epilepsy seem to be unaware of missed doses, indicating the need for pill dispensers and reminding/educational interventions [21].

Self-efficacy, defined as the confidence to carry out a behavior necessary to reach a desired goal [19] an important concept in self-management. Self-efficacy and changes in self-efficacy are associated with future health status and it appears that enhanced self-efficacy is at least one of the mechanisms responsible for the improvements in health status demonstrated by those attending self-management programs [22-24]. Working within the field of epilepsy, Pramuka et al. [20]. piloted a psychosocial self-management program for epilepsy and observed a positive correlation between self-efficacy and quality of life. In addition, Amir et al. [18] emphasized the possibility of increasing quality of life among patients with epilepsy by counseling and treatment aimed at increasing their self-efficacy. However, the Managing Epilepsy Well network recently concluded that too few self-management programs exist in general and that there are too few evidence-based programs available [25].

This study will therefore evaluate the feasibility and (cost-)effectiveness of a multi-component intervention (MCI), which combines a self-management/education program with e-Health interventions, aiming to improve self-efficacy and concordance in people with epilepsy, in comparison with care as usual (CAU). Hence the MCI focuses on increasing patients’ understanding of their medical regimens, and on providing skills and tools to strengthen self-management and communication between patient and healthcare professional and increase adherence (as a proxy for concordance).

## Methods

This study will consist of three parts, each with its own research questions:

I. Clinical effectiveness
  1. Is MCI, in comparison with CAU, more effective in terms of self-efficacy and other patient-reported outcomes (self-efficacy, adherence, decrease in seizure frequency & severity, side effects of AED, controlling depression/anxiety, proactive coping, improved quality of life, and societal costs)?
  2. Does the MCI have a clinical superiority over CAU in terms of a better adherence to AEDs?
II. Economic evaluation
  3) What is the cost-effectiveness and the cost-utility of the MCI in comparison with CAU from a societal perspective?
III. Process evaluation
  4) Has the MCI been delivered according to protocol? And if not, what are the reasons for protocol deviation?
  5) What are the experiences and opinions of patients, caregivers and professionals regarding the MCI?
  6) To what extent has the MCI impacted concordance among patients (i.e. do patients understand why it is relevant to take the AEDs, and has the MCI impacted shared decision making by means of consensual agreement between patient and doctor regarding the medical regimen?)

## Design

**Figure 1:**
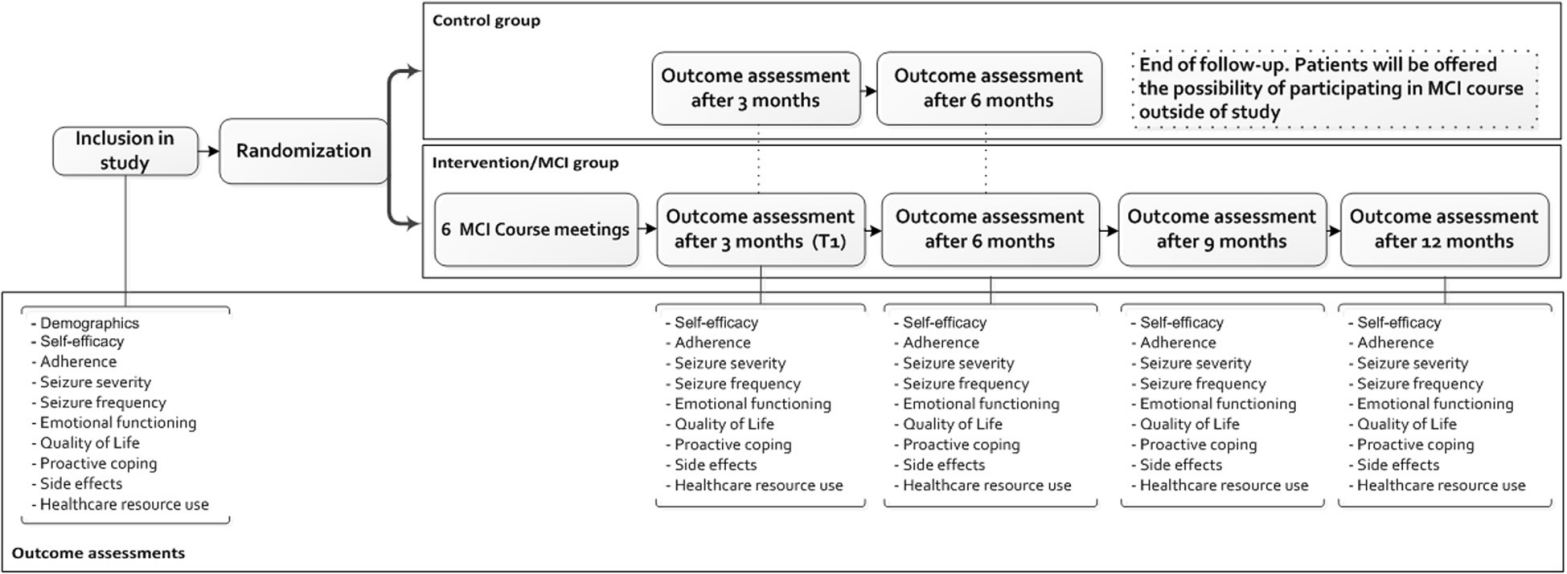
**Schematic representation of study design.**

A randomized controlled trial (RCT) with two parallel groups will be conducted to compare the MCI with a control situation in epilepsy patients. Patients assigned to the intervention group will attend the MCI consisting of five weekly sessions and one booster session. The control group will receive CAU as naturally as possible. The follow-up of patients in the intervention group will be 12 months and the follow-up of patients assigned to the control group will be 6 months, after which patients in the control group have the opportunity to receive the MCI outside the study (see Figure 1). The study has been approved by the Ethics Committee of University Hospital Maastricht, the Netherlands.

### Study population

One hundred eligible epilepsy patients will be included in the study. Eligible patients are adults aged 18 or over, living at home, diagnosed with epilepsy and using AEDs, who understand the Dutch language, and are willing and able to use e-Health devices belonging to the MCI. Patients will be excluded if they are not able or willing to function in group activities or when it is expected, on the basis of clinical judgment, that patients are not able to comprehend topics discussed within the MCI (i.e. patients with cognitive deficits).

### Setting and recruitment

This study is a collaboration between Maastricht University and the epilepsy center Kempenhaeghe (KH). The study will be conducted at the outpatient clinics of KH. The first MCI will be offered at the outpatient clinic at the main location of KH (Heeze), after which the MCI will be offered at other outpatient clinics (i.e. Nijmegen and Maastricht). From April 2014 onwards, neurologists and nurse practitioners (NP) will recruit possible candidates for participation during consultations at the outpatient clinics of KH. Furthermore, a press release will be published in national epilepsy magazines and on social media, and patient information leaflets will be distributed to patients in the outpatient clinics of KH. In the press release patients are invited to send their contact information to the researchers.

When patients show interest in participating, an information meeting will be scheduled with one of the researchers (LAML or BFMW), either by phone or face-to-face, in which patients will have the opportunity to ask questions and in which inclusion and exclusion criteria will be checked either by the researchers during the meeting or afterwards in consultation with clinicians. During the meeting, patients are informed about the study and that they will be randomly assigned either to the intervention or control group. After one week researchers will contact patients who have received information and want to take part in the study to plan a visit. During this visit an informed consent form will be signed by the patient and the researcher, and patients will be allocated randomly to either the intervention or control group.

### Sample size

The primary outcome variable of this study will be self-efficacy as measured on the Epilepsy Self-Efficacy Scale 33 (ESES) [26]. In a previous pilot study exploring the effects of a psychosocial self-management program for epilepsy patients, with self-efficacy as the primary outcome, the difference between groups was approximately 10 points, with a standard deviation of 7 points on the ESES [20]. Assuming that alpha = 0.05 and power p = 0.90, a minimally detectable difference of 5 points between the intervention and control groups, we will need to include 42 patients per group. Based on a dropout rate of 20% we intend to include 50 patients in each group.

### Randomization

Patients will be randomized to the MCI (intervention group) or to the control group, in which patients will be given the opportunity to attend the MCI outside of the study after six months of follow-up. For parallel provision of both groups (intervention & control), two blocks of five patients are needed at the moment of randomization. Patients will be assigned to the intervention group or the control group by means of block randomization, using blocks of 10 patients. An assistant who is not involved in the treatment nor in the trial will execute the procedure with a randomization program (www.randomization.com).

### Multi-component intervention (MCI)

Self-management is a process in which patients take responsibility for changing their health behavior by acquiring knowledge about their disease and treatment, and by managing symptoms as well as the physical and psychosocial consequences of the disease [27].

This definition is a compilation of a broad range of definitions about self-management, combining physical functioning and outcomes with knowledge and the psychosocial consequences of disease. Our MCI is based on this idea. In order to manage symptoms or the physical and psychosocial consequences, we will try to provide patients with knowledge about self-monitoring (by use of e-health tools) and risk management. In addition, in order to change behavior, emphasis will be put on proactive coping, concordance and goal-setting.

Patients will have the opportunity to bring one of their relatives/friends for social support, based on the fact that self-management is not only supported by healthcare providers, but also by the people surrounding persons with chronic diseases [27]. The intervention is based on the self-management intervention offered in the Restore4Stroke study [28] but was adjusted to make it suitable for epilepsy patients. The final version of the intervention was developed in agreement with experts in the field of self-management, coordinators of the Restore4Stroke study, clinicians, and representatives of the Epilepsy Association of the Netherlands (EVN, Epilepsie Vereniging Nederland).

The intervention will be explained in a detailed protocol for the NP offering the intervention in the outpatient clinic and in a workbook for patients and one of their relatives/friends. In addition, NPs will receive training beforehand on motivational interviewing (MI), as a technique to empower patients to set their own sustainable goals and look into conflicting beliefs. MI is defined as “a collaborative, person-centered form of guiding to elicit and strengthen motivation for change” [29]. MI focuses on exploring and resolving ambivalence and centers on motivational processes within the individual that facilitate change [30].

### Group sessions

The MCI is offered as a group treatment to groups of 5 patients and additional family members and/or friends. The MCI will last 9 weeks. During the first 5 weeks, group sessions will take place once a week, followed by a booster session 3 weeks later. The group sessions last 2-2.5 hours and are led by an NP with experience in working with epilepsy patient groups. The first session is aimed at providing information about the MCI, including materials, and getting to know the other participants and therapists. During the next sessions, participants will practice with the five stages of proactive coping defined by Aspinwall & Taylor [31]. These five stages are (1) resource accumulation, (2) recognition of potential stressors, (3) initial appraisal, (4) preliminary coping efforts, and (5) elicitation and use of feedback concerning initial efforts.

The model will be applied by the patients to three fixed epilepsy-related themes. The first theme will be self-monitoring and self-monitoring (e-Health) tools. The other two themes will be risk-management and shared decision-making/concordance.

Each group session will have the same basic structure. The sessions will begin by looking back at goals set in the previous meeting and how things have worked in the last week. Next, the theme of the session will be introduced. Patients and caregivers will be invited to share their beliefs, emotions and experiences with regard to the theme. Subsequently, patients and caregivers will formulate their own action plan in order to attain a goal relevant to the theme. Patients will be instructed to keep their goals feasible, and group members will give feedback on the quality of the goals in terms of concreteness and attainability. They will help each other to recognize additional conditions and barriers which need to be addressed. After the feedback discussion, patients and caregivers will formulate their final plan.

### e-Health

The e-Health tools used in the intervention consist of 3 elements: 1) the Medication Event Monitoring System (MEMS; Aardex Ltd., Switzerland), 2) a smartphone application, and 3) an internet accessible patient database. The MEMS caps are electronic caps that fit on standard pill bottles. They register the date and time every time the pill bottle is opened. In addition, the MEMS of the intervention group will include an LCD-screen which provides feedback on the number of times the bottle is opened on a particular day. These data can be downloaded from the MEMS cap by the researchers with a communication device and a computer. A computer program will then present the data in simple plots which can be used to provide feedback about behavior, and to identify sub-optimal adherence patterns [32]. Feedback will be provided during the MCI and during each follow-up visit. The smartphone application (“Eppy”; Epilepsy Foundation, the Netherlands) is designed to register seizure frequency and other facts for persons with epilepsy, in order to provide data which can influence the management of epilepsy. “Eppy” can be downloaded at no charge from the App store (Apple Inc., USA) or from the Google Play store (Google Inc., USA). The application provides patients with the opportunity, among others, to keep a seizure diary, to set reminders for the intake of medicines and an alarm mode in which a text will be shown for bystanders in case of a seizure. Data gathered with “Eppy” can be synchronized to an internet accessible database, which gives an overview of all clinical events registered by the patient. This internet website is owned by the patient, who can allow healthcare professionals to access it for clinical and research purposes.

### Booster session

During the booster session the NP will rehearse goal setting themes and other themes discussed in the group sessions. Patients and caregivers will have the opportunity to discuss their experiences related to their goals and other aspects of the intervention.

### Control group

The control condition will be a control condition with unrestricted access to CAU. Care will not be intensified after enrollment. As this will be a pragmatic trial, CAU will not follow a standardized protocol. However, medical support will be documented in the electronic patient file of each patient. Medical support provided in the control group might be variable, but is expected to be in agreement with the standard epilepsy guidelines (i.e. preference for mono-drug therapy, a monitoring visit every 4 months by neurologist or epilepsy nurse and change or addition of medication if the first AED is ineffective) [33]. The control group will receive MEMS but without feedback about their behavior. The MEMS will be read only at the end of the follow-up as a way to measure adherence and will not include an LCD-screen.

## Clinical effectiveness

To assess the (cost)-effectiveness of the MCI, outcome assessments will be done at baseline and after 3, 6, 9, and 12 months (9 and 12 months applicable only for intervention group). A detailed overview regarding the outcome assessments can be found in Table 1. The following outcomes are defined:

I. Primary outcomes
  1. Epilepsy Self-Efficacy Scale-33 items (ESES). The ESES is a 33-item scale that measures different aspects of efficacy within the self-management of epilepsy. The items represent three dimensions of self-management: medication management, seizure management, and general management including safety and health [26]. Items are rated on an 11-point Likert rating scale, ranging from 0, “not at all certain I can do”, to 10, “very certain I can do” [34]. The total possible scores for the ESES range from 0 to 330. Higher scores correspond to higher levels of confidence in the ability to manage epilepsy.
II. Secondary outcomes
  2) Adherence, used as a proxy for concordance, which will be determined using: 1) MEMS. Electronic monitoring, such as MEMS has been proposed as a possible “gold standard” for medication adherence measurement [15,35], and 2) the Medication Adherence Rating Scale (MARS-5), which measures self-reported adherence. The MARS-5 contains 10 items, each of which has to be answered with yes or no. Hence, the final score on the MARS-5 ranges from 0 to 10, in which a higher score represents better adherence.
  3) General self-efficacy, which will be determined using the Dutch adaptation of the general Self-Efficacy Scale (GSES). The GSES consists of 10 items assessed on a 4-point scale, ranging from ‘totally wrong’ to ‘totally true’. The scale was designed to assess self-efficacy, i.e., the belief that one’s actions are responsible for successful outcomes. The scale scores for each question ranges from 1 to 4 resulting in an overall score between 10 and 40. Higher scores indicate patient’s stronger belief in self-efficacy [36,37].
  4) Seizure frequency will be determined using a short questionnaire regarding seizure frequency covering the past 4 weeks. The questions focus mainly on the number of seizures, whether the person documents his/her seizures and in what way the person documents his/her seizures.
  5) Seizure severity, which will be determined using the National Hospital Seizure Severity Scale (NHS3). The NHS3 lists seven seizure-related factors and generates a score from 1 to 27, in which a higher score represents a more severe seizure [38].
  6) Adverse events of AED, which will be determined using the SIDe-effect of the AntiEpileptic Drugs questionnaire (SIDAED). The SIDAED consists of 46 items regarding possible AED-related complaints. These items form 10 categories: general CNS, behavior (increased irritability), depressive symptoms, cognitive function, motor problems and co-ordination, visual complaints, headache, cosmetic and dermatological complaints, gastrointestinal complaints, and sexuality and menses [39]. For each item the patient rates the severity of the complaint on a four- point Likert scale (no problem, mild, moderate, or serious problem). In addition, the duration of the complaints is scored (a few weeks, months or half a year or longer). The SIDAED ranges from 0 to 138, in which a higher score indicates more severe/frequent side-effects [39].
  7) Depression/Anxiety, which will be determined using the Hospital Anxiety and Depression Scale (HADS). The HADS has a total of 14 items, each scored on a scale of 0-3, with 3 indicating higher symptom frequencies. Scores for each subscale (anxiety and depression) can range from 0-21 and scores for the entire scale (emotional distress) range from 0-42, with higher scores indicating more distress [40].
  8) Self-rated proactive coping, which will be determined using the Utrecht Proactive Coping Competence Scale (UPPC). A total of 21 items are assessed on a 4-point scale ranging from ‘not competent at all’ to ‘very competent’. Total scores are calculated by averaging the 99 individual item scores. Higher scores on the UPCC indicate higher levels of perceived proactive coping competencies [41].
  9) Disease-specific quality of life will be measured with the Quality Of Life in Epilepsy-patient-weighted (QOLIE-31-P). The QOLIE-31-P consists of 38 items assessing 7 domains of epilepsy: Seizure Worry, Overall QOL, Emotional well-being, Energy-Fatigue, Cognitive Functioning, Medication Effects, Social Functioning and an Overall Score. In addition, for each domain, questions regarding how much distress a person feels about problems and worries related to epilepsy are included [42]. Each domain is scored on a scale ranging from 0 to 100. Afterwards a final score can be calculated using weights derived from the amount of distress related to each domain. The final score ranges between 0 to 100, in which higher values indicate a better Quality Of Life [43].
  10) Generic Quality Of Life will be assessed with the EuroQoL 5 dimensions 5 levels (EQ-5D-5L). The EQ-5D-5L consists of five dimensions: mobility, self-care, usual activities, pain/discomfort, anxiety/depression, each of which can have one of five responses [44,45]. Each health state will be valued using the Dutch tariffs, which will result in utilities on a scale from 0 to 1. Utilities derived from the EQ-5D-5L will be used in calculating the quality adjusted life years (QALY) by multiplying the time spent in a health state by the utility assigned to that health state.
  11) Societal costs will be measured retrospectively with the a Medical Cost Questionnaire (MCQ), an adapted version of the Trimbos/iMTA questionnaire for costs associated with psychiatric illness [46] and the Productivity Cost Questionnaire (PCQ), each covering 3 months.

**Table 1:** **Overview of measurements per time point**

**Outcomes patients**	**Instrument**	**Short term**	**T0**	**T1**	**T2**	T3*	T4*
Demographic and clinical characteristics	-	**-**	X	**-**	**-**	**-**	**-**
Self-efficacy	Epilepsy Self-efficacy Scale – 33 items	ESES	X	X	X	X	X
General self-efficacy	Generic Self-efficacy Scale – 10 items	GSES	X	X	X	X	X
Adherence	MEMS Medication Adherence Scale	MARS 5	X	X	X	X	X
Seizure frequency	Questionnaire seizure frequency	-	X	X	X	X	X
Seizure severity	National Hospital Seizure Severity Scale	NHS3	X	X	X	X	X
Emotional functioning	Hospital Anxiety and Depression Scale	HADS	X	X	X	X	X
Quality of life	Quality Of Life in Epilepsy	QOLIE31P	X	X	X	X	X
	Generic quality of life	EQ-5D-5L	X	X	X	X	X
Proactive coping	Utrecht Proactive Coping Competence	UPCC/PCI	X	X	X	X	X
Side effect	Side effects of Anti-epileptic Drugs	SIDAED	X	X	X	X	X
Healthcare resource use	Medical Cost Questionnaire	MCQ	X	X	X	X	X
	Productivity Cost Questionnaire	PCQ					

T0 = baseline outcome assessments; T1 = Outcome assessments after 3 months; T2 = Outcome assessments after 6 months; T3 = Outcome assessments after 9 months; T4 = Outcome assessments after 12 months.

*Outcome assessment applicable only for intervention group.

## Economic evaluation

The trial-based economic evaluation will be performed from a societal perspective and will consist of a cost-effectiveness analysis (CEA) and a cost-utility analysis (CUA). Outcomes of interest for the CEA and the CUA will be self-efficacy as assessed by the ESES, and generic quality of life as assessed by EQ-5D-5L. We distinguish four cost categories: intervention costs, healthcare sector costs, costs for the patient and family, and productivity costs. Intervention costs will be defined as all costs related to the MCI including travel costs, personal costs, material costs, costs of e-Health tools (i.e. MEMS) and housing costs. Healthcare and patient costs will be estimated using a questionnaire regarding healthcare resource utilization and productivity losses.

Resource use and outcomes are measured at the same time points mentioned in the effectiveness study: at baseline, 3 months, 6 months, 9 months, and 12 months in the intervention group and at 3 months and 6 months in the control group. A comparison between MCI and CAU will be made in terms of incremental costs and incremental effects. The time horizon will be 12 months (trial-based economic evaluation). To measure the use of health care resources, including all activities related to epilepsy, we gather data for each patient at baseline and at a follow-up of one year. Cost calculations will be based on the Dutch guidelines for cost calculations in healthcare [47].

## Process evaluation

Process evaluation will be performed to assess whether the MCI was delivered according to protocol, to examine the experiences and opinions of patients, caregivers and professionals regarding the MCI, and to determine to what extent the MCI has impacted concordance among patients. The process evaluation will be performed according to the framework provided by Saunders et al. [48]. This framework consists of a stepwise approach in which important characteristics for the process-evaluation plan are identified along seven basic components, namely: fidelity (quality), dose delivered (completeness), dose received (exposure), dose received (satisfaction), reach (participation rate), recruitment and context.

We will use a mixed methods design in which both qualitative and quantitative data will be collected. The qualitative part will consist of observations during several group sessions over time. After each observation, a short interview will be held with the group leader(s) in which the group leader can reflect on his/her opinion regarding the session. During the last (sixth) group session of every MCI-group, a short evaluation form will be handed out to patients in which they will be asked to rate different aspects and themes of the MCI on a 7-point Likert scale. Furthermore, at the end of the study, focus groups will be held consisting of patients included in the study. The selection of participants will be based on maximal variation to get as many perspectives as possible. Participants will be selected based on age, sex and severity of seizures and effectiveness of the intervention (to compare patients for which the intervention was successful versus unsuccessful). Focus groups will be conducted using a semi-structured questionnaire covering the topics identified in the framework provided by Saunders et al. [48].

## Analysis

### Clinical effectiveness

Baseline characteristics will be described, and differences between groups at baseline will be studied using t-tests or chi-square tests where appropriate. All statistical procedures will be conducted based on both the intention-to-treat principle and on actual participation in treatment (i.e. effectiveness analyses) and will be performed using SPSS statistics 22.0 (SPSS, IBM, Corporation, Chicago, USA). Missing data will be handled using SPSS missing value analysis on item level. Completely missing measurements will be handled using multiple imputation. To evaluate outcomes, change scores will be calculated and compared between the groups after treatment. Multi-level analyses will be performed with measurements (T0, T1, T2, T3 and T4) within the subjects’ factor and group and between subjects’ factors to account for the nested structure of the data. Data from the control group will be extrapolated to 12 months. Post hoc analyses will be performed in case of significant effects. Baseline differences will be corrected by inclusion of covariates in the analyses. A 2-sided significance level of 0.05 will be used as a threshold to determine whether differences are statistically significant.

### Economic evaluation

Costs calculation will be performed according to the bottom-up approach, based on a detailed inventory of all cost items. Standardized cost prices from the Dutch manual for costing will be used in the calculations or (if not available) calculated mean cost prices, according to providers, will be used [47]. To determine the costs of drugs, the Dutch consumer reimbursement price for medication will be used. Productivity costs of the patients will be estimated with the friction cost method. Costs will be indexed for the year 2015.

As cost data are normally skewed, parametric tests are mostly not suitable. Hence non-parametric bootstrapping (1000 times) will be used to test for statistical differences in costs between groups and to investigate the uncertainty around the costs. Bootstrapping will be done using Microsoft Excel (Excel, Microsoft Corporation, Washington, USA). Bootstrap replications will be used to calculate 95% confidence intervals around the costs, based on the 2.5th and 97.5th percentiles. For the cost-effectiveness analysis, the corresponding incremental cost-effectiveness ratio (ICER) will be expressed as incremental costs per increased adherence and incremental costs per self-efficacy (ESES). In the cost-utility analysis, the ICER will be expressed as the incremental costs per QALY gained. QALYs will be calculated using the area under the curve method. All bootstrapped ICERs (5000 times) will be presented in a cost-effectiveness plane to determine the robustness of the ICER. To determine the probability that the MCI is cost-effective given a certain ceiling ratio, a cost-effectiveness acceptability curve will be constructed. In addition, one-way and multi-way sensitivity analysis will be performed on the most important cost parameters.

### Process evaluation

Quantitative data will be analyzed by descriptive statistics (i.e. frequencies, mean and median), Chi square tests, and ANOVA. Results from open-ended questions included in the questionnaires, focus group interviews and interviews will be categorized to identify relevant themes.

## Discussion

This study will determine the (cost-)effectiveness and feasibility of the MCI to improve the management of epilepsy in adult patients and increase self-efficacy and concordance regarding AED. The MCI is designed to stimulate self-management skills and the awareness of patients with epilepsy in combination with the use of e-Health interventions. In times of budget constraints, MCI could be a valuable addition to current healthcare provisions for epilepsy, as it is expected that higher concordance and self-efficacy will result in reduced healthcare resource use and an increased QOL.

Cooperation between research and practice is the key strength of this project, enabling the intervention to be studied in a natural environment; the project will facilitate further implementation of the multi-component program into the standard practice of KH and of other institutions. Both professionals and patients played important roles in the development of this program and will also be involved during the evaluation and implementation of the MCI program.

One of the limitations of our study design is that, in case the MCI is shown to be effective, it will be difficult to identify what components contribute to this effectiveness. For example, it could be that only the increased attention from nurses or the adherence monitoring contribute to improvements in the patients’ health status. However, we believe we have included a large variety of outcome assessments which, altogether, form a broad view of the (possible) effectiveness of MCI.

To the best of our knowledge, there are currently no validated questionnaires available to examine concordance. However, as this study is striving to improve ‘concordance’ and not only ‘adherence’, it is assumed that any increase in adherence by the MCI is partly explained by increased concordance (i.e. due to the educational content in the MCI). In addition, special attention will be paid to concordance during the process evaluation. Accordingly, this study is aimed at making patients their own provider of health care, thus shifting epilepsy management from professionals to self-care by patients equipped with appropriate skills and tools.
